# Bending mechanics test and parameters calibration of ramie stalks

**DOI:** 10.1038/s41598-023-35469-x

**Published:** 2023-05-29

**Authors:** Shuning Si, Bin Zhang, Jicheng Huang, Cheng Shen, Kunpeng Tian, Haolu Liu, Yuanyuan Zhang

**Affiliations:** Nanjing Institute of Agricultural Mechanization, Ministry of Agriculture and Rural Affairs, Nanjing, 210014 China

**Keywords:** Mechanical engineering, Structural materials, Biophysical methods, Structure determination

## Abstract

Research and development of ramie harvesting equipment is a key link to revitalize ramie industry, problems such as the tendency of stalks to tangle and clog the machine are very problematic, seriously affect the quality and fluency of the harvester. The structure of ramie stalk is complex, and the mechanical properties of each component vary greatly, collision between stalk and machine creates complex stress relationship. By building a finite element model, it is possible to analyze the stress state of the stalk during bending from a microscopic perspective, and to analyze the complex stress–strain situation within the stalk. The purpose of this paper is to establish a standard ramie stalk bending finite element model to provide a theoretical basis for the subsequent kinematics and dynamics. Firstly, material experiments were carried out on ramie straw. The structural and mechanical parameters of the straw components were obtained through measurement and calculation tests, and the force–deformation curves for straw bending were obtained. Bending finite element simulations were carried out on the basis of mechanical tests, and the parameters such as dynamic friction coefficient, wood Poisson's ratio and bast Poisson's ratio were determined by the central combination design. Then established an accurate bending finite element simulation model of ramie stalk, the accuracy of the model was verified at the end. In this paper, the key parameters of the ramie stalk model were calibrated through a combination of material tests and simulations. All parameters of the ramie stalk model were finally obtained, and the bending mechanical properties of the ramie stalk were analysed by applying finite element analysis. This bending mechanics simulation model can be used for kinematic and dynamics simulation analysis of conveying and baling to provide a theoretical basis for the structural design of the harvester. The methods explored here can be applied to other slender straw crops.

## Introduction

Ramie is a traditional economic crop in China, it has a high value for textile, medicinal, fodder and soil conservation purposes, its tender stalks and leaves are rich in protein, amino acids, vitamins, minerals and other nutrients, making it a high-quality vegetable protein feed material^1,2^. Manual cutting is still used, the working environment is harsh, with high labor intensity and low work efficiency. Overcoming the problems of stalk damage and winding is a key step to realize the mechanized ramie harvesting equipment^3,4^.

Su et al.^5–7^ measured the mechanical properties parameters of the xylem, phloem and other components of ramie stalk through mechanical tests such as tensile, compression and bending. But the poisson's ratio of ramie fibre and wood could not be obtained directly from the tests. Wang et al.^8^ calibrated the cutting finite element model of wild chrysanthemum stalks during harvest, screened the significantly influential test factors by P-B tests, obtained the optimal parameters by central combination design, and conducted cutting force simulations to verify the accuracy of the parameters. Hou et al.^9^ studied the bending mechanical properties of different varieties of castor stalks at various levels of factors, the effect of fiber duct content on the flexural strength of stalk was studied by establishing a finite element model. Xue et al.^10^ used ANSYS to study the bending deformation and stress distribution of cassava hemp stalks under ultimate load. Yuan et al.^11^ used the finite element software ANSYS/LS-DYNA to establish a model of wheat stalks, simulate and analyse the bending collapse process of wheat stalks under rectangular wind load. The material of plant stalks and their derivatives needs to be harvested and processed, Studying the deformation behavior of the stalk and its interaction with the machine (due to the mechanical loads imposed by the machine) is critical for process optimization and yield efficiency. Darshil U. Shah et al.^12^ dialectically analyzed various methods for testing the structural properties of stalks, He believes that stalk morphology and other structural characteristics are important factors to consider when designing test protocols. The mechanical parameters and damage forms of the stalks under different test conditions were obtained through mechanical testing of the stalks.

Xu Xin et al.^13^ used SolidWorks Simulation to simulate the mechanism of the ramie peeling machine; Yan Kemang et al.^14^ used ABAQUS software to numerically simulate the bast and xylem of the ramie stalk. However, all these researchers simply brought the mechanical parameters of each component of the stalk into the model, without verifying the reliability and standardization of the overall mechanical parameters. Ramie stalks have a complex structure, and the mechanical properties of each component vary greatly. Simply bringing the parameters of each component into the model cannot guarantee that the mechanical properties of the stalk as a whole are consistent with reality. Ramie fiber is an important raw material for composite materials^15^. Djafar et al. explored the influence of the number and thickness of ramie fiber layers on the flexural strength and tensile strength of the overall material^16^.

**Figure 1:**
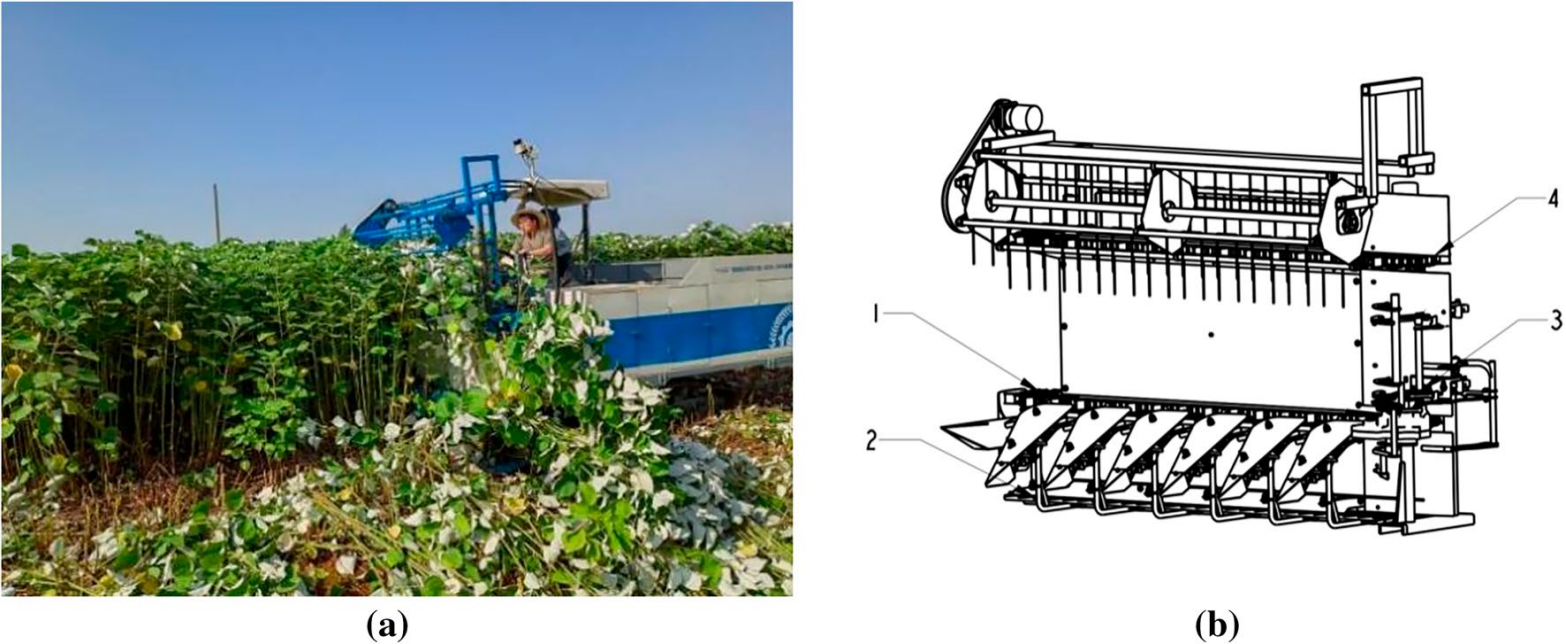
Ramie harvester. (**a**) Field harvest, (**b**) harvester structure. (1) Bottom chain, (2) cutter, (3) baling device, (4) second row chain.

The basic theoretical research on ramie harvesting mechanization technology is very backward. This article fills the gap in the research on the basic physical and mechanical properties of ramie, and also advances the process of applying simulation technology in the field of ramie harvesting.Ramie is prone to breakage during harvesting and baling, and the flexible ramie fibres can easily become entangled with the mechanism, seriously affecting the overall work quality and efficiency of the equipment^17,18^. The combination of material tests and simulation tests to analyze the bending mechanical properties of the stalks, establish a standard ramie stalk finite element model, these works can improve the reliability of subsequent simulation studies, provide a theoretical basis for the design and improvement of harvesting equipment.As shown in Fig. 1, the structure and test of the ramie harvester.

## Materials and methods

**Figure 2:**
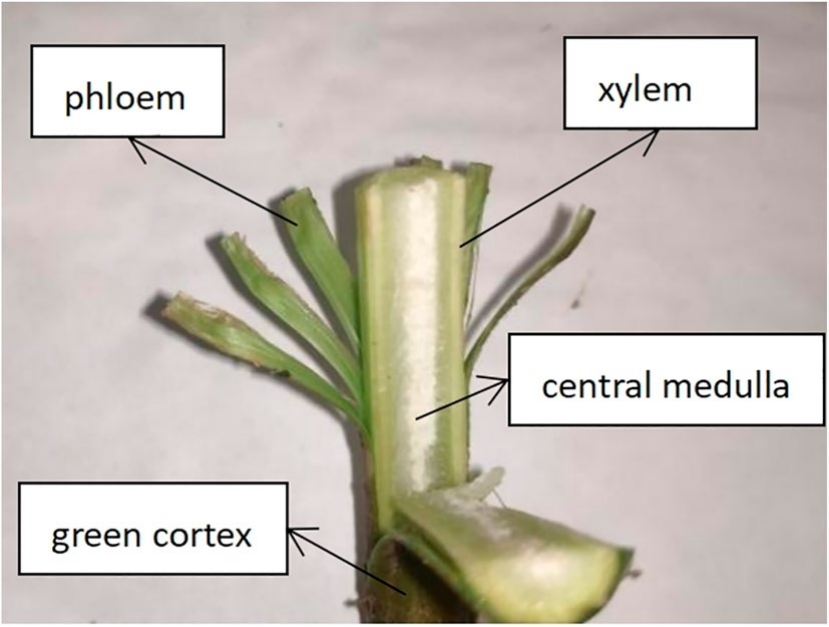
Schematic diagram of the structural composition of the ramie stalk.

The aspect ratio of the ramie stalk reaches 200:1, the basic structure of the ramie stalk is shown in the Fig. 2. The central pith is foamy, its tissue is loose; the wood is hard but easily to fold; the fresh cortex includes the bast and the green cortex, the phloem contains ramie fibres, soft and tough. The raw hemp that can be obtained by drying the phloem, which is a high quality raw material for the textile industry and can create considerable economic benefits.

Stalk is a living solid material, and its bending mechanical properties are often affected by moisture content, maturity, temperature, deformation rate and other factors. This puts forward higher requirements for the harvesting and processing efficiency of ramie.

### Experimental study on bending mechanical properties of ramie stalk

#### Test Preparation

1. Specimen collection
  The ramie material is "Zhong Zhu-I" cultivated at the Xianning Ramie Experiment Station of the National Hemp Industry System. The ramie was harvested on May 25 and is in its harvesting stage. The stalks were randomly selected from ramie plants with straight, well-grown stalks, free from pests and diseases, and no damage. Store the prepared ramie stalks in an airtight container and refrigerated.The collected stalks are shown in Fig. 3.

**Figure 3:**
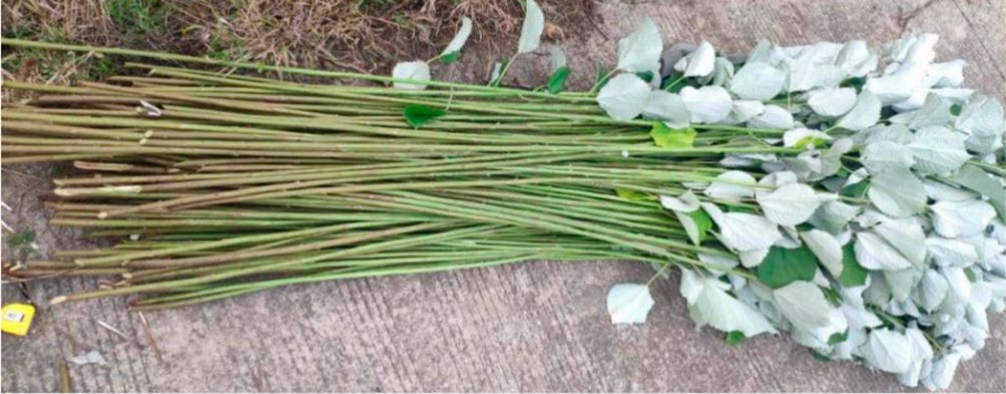
Stalk collection.
2. Specimen preparation and parameter measurement
  The average length of the stalk specimens for the bending test was 180 mm, and ten specimens were taken for each set of tests. Specimen preparation: the sampling position for the "loading position" factor test is 10–28 cm, 60–78 cm and 110–128 cm from the ground. A diagram of the sampling site is shown in Fig. 4. The stalk in position ① corresponds to the cutting part of the ramie harvester, which also often breaks and causes the ramie to fall over in the field. The height of ② and ③ is the height of the lower and upper chains of the ramie harvester's clamping conveyor. The prepared test samples are shown in Fig. 5.

**Figure 4:**
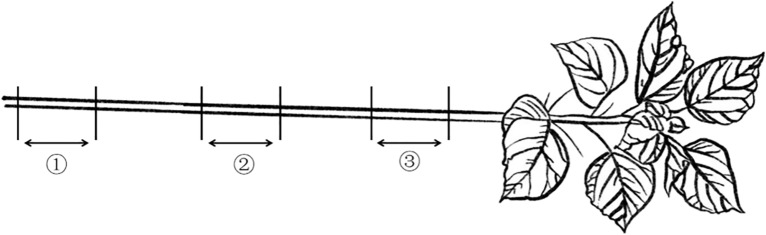
Schematic diagram of the sampling position.

**Figure 5:**
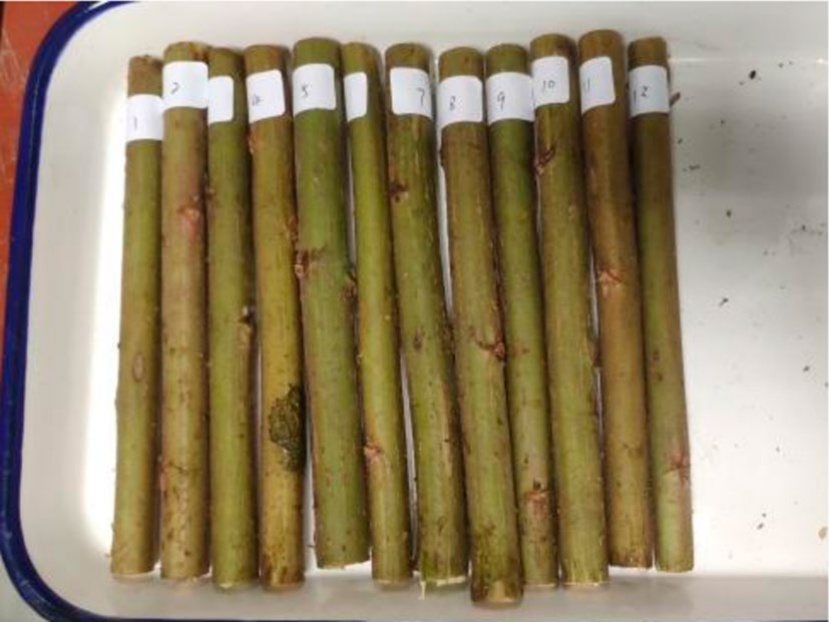
Sample preparation.
3. Measurement of basic parameters
  Measure multiple sets of data and take the average, and obtain length parameters using tools such as vernier calipers; the moisture content of the stalks was determined using a blast dryer and an electronic balance; cylinder and balance measurements were used to calculate the density of the stalks through the weighing method; the coefficient of static friction between the stalks and steel was measured using the ramp method to obtain the results of the basic physical parameters of the stalks (Table 1).

**Table 1:** Basic physical parameters of ramie stalk.

Parameter type	Value	parameter type	Value
Whole plant height	231.40 cm	Whole plant weight	198.30 g
Trunk height	149.70 cm	Trunk weight	145.50 g
Root diameter	15.70 mm	Inner diameter of root	7.40 mm
Top diameter of main stalk (at 150 cm)	10.90 mm	Inner diameter of top of main stalk (at 150 cm)	6.20 mm
Average density of the main stalk	0.97 g/cm^3^	Range of variation in moisture content	80.7–82.12%
Fresh cortex to stalk ratio	21.42%	Centroid height	103.10 cm
4. Test equipment
  The mechanical property testing equipment adopts SUNS-UTM6503 microcomputer-controlled electronic universal testing machine (shown in Fig. 6), using the bending mechanics test module, the test standard range is 5 kN, the measurement accuracy of the sensor is ± 0.1%. DGF30/7-1A electric blast dryer, HANGPNG- FA1004 electronic balance, basic parameters measurement tools include vernier calipers (accuracy 0.02 mm), measuring cylinders, etc.

**Figure 6:**
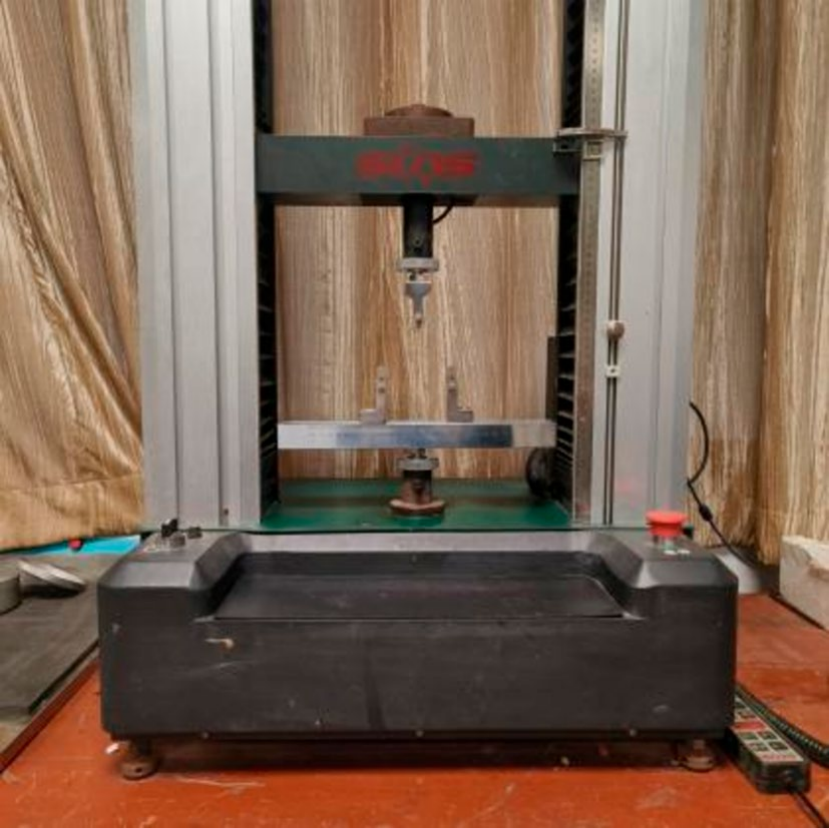
Test bench.

#### Ramie stalk bending test protocol

The ground where the ramie grows is the origin of the axis, and the axis is established along the direction of stalk growth. The distance of the loading point from the origin of the axis is the loading position, and the moisture content of the specimen at the end of the test is determined and verified. The three-point bending method was used to carry out a single-factor bending test on the stalk. Bending test method refer to GB/T 1456-2021 metal bending mechanical properties test method^19^.

**Figure 7:**
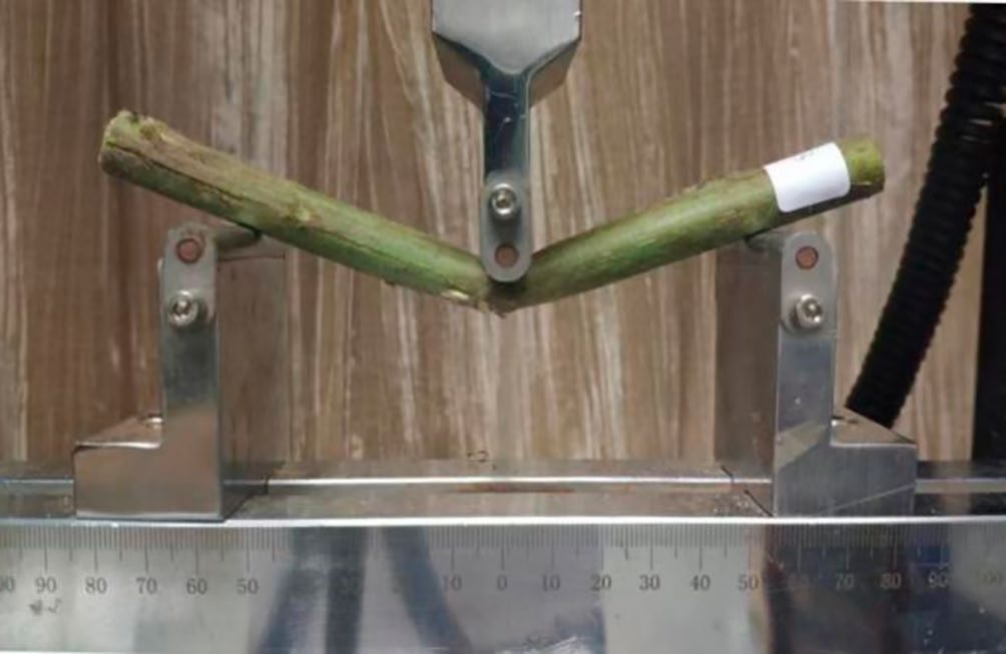
Bending test.

The factor levels are shown in Table 2. The geometric parameters of the specimen were measured using vernier calipers for recording; the specimen was placed between the support and indenter of the three-point bending test fixture with a span distance L of 100 mm and a start-up preload force of < 5 N. Ten sets were repeated at each level, for a total of 30 sets of tests. The bending test is shown in the Fig. 7.

**Table 2:** Single-factor level table.

Number	X_1_	X_2_	X_3_
Loading position X (cm)	19 cm	69 cm	119 cm

#### Principles of ramie bending calculation

1. The radial three-point bending test was carried out on the ramie sample stalk by the universal material testing machine. The radial shear stiffness of the stalk is obtained with the help of the calculation formula of the shear stiffness of the circular tube (formula (1)) and the calculation formula of the shear modulus of the circular tube (formula (2)), the radial shear stiffness U and bending modulus of elasticity G of the stalk were obtained.
  1 $$\mathrm{U}=\frac{{\Delta {\rm p}}\cdot \mathrm{L}}{4(\mathrm{f}-\frac{{\mathrm{f}}_{1}\cdot \mathrm{L}}{3\mathrm{a}})}$$
  where U is shear stiffness (N), Δp is elastic phase load increment (N), L is span distance (mm); f is mid-span deflection increment (mm); f_1_ is epitaxial deflection increment (mm); a is epitaxial length (mm).
  2 $$\mathrm{G}=\frac{2\mathrm{U}}{\uppi /4({\mathrm{D}}^{2}-{\mathrm{d}}^{2})}=2.564\frac{\mathrm{U}}{{\mathrm{D}}^{2}-{\mathrm{d}}^{2}}$$
  where G is bending modulus of elasticity, (MPa); U is shear stiffness (N); D is specimen outer diameter (mm); d is specimen inner diameter (mm).
2. The volume ratio of phloem V_1_.
  3 $${V}_{1}=\frac{4Dh-4{h}^{2}}{{D}^{2}-{d}^{2}}$$
  where D is specimen outer diameter (mm); d is specimen inner diameter (mm); h is phloem thickness (mm).

### Calibration of bending mechanics model parameters for ramie

In the study of mechanical equipment related to the harvesting and processing of plant stalks and their derivatives, understanding the overall deformation of the stalk and its interaction with the machine (loads from the machinery) is critical for structural optimisation and efficiency improvement. Finite element simulation has the advantages of flexibility, efficiency and low cost. Based on mechanical tests of stalk bending, the force response of ramie stalks during bending was investigated by studying the microscopic deformation of the stalks to explain the observed mechanical behaviour.

#### Stalk model simplification principle

Based on the establishment of the constitutive relations of the physical models of wood and sugarcane,the ramie stalk is abstracted as a composite material composed of bast fiber and xylem, and its performance is homogeneous, linear elastic. The parameters in the constitutive relation are determined by bending tests and consulting data^8–10,20^. Based on the assumptions of ramie geometry, ramie has an axial rotation and the existence of orthogonal symmetrical planes gives it the characteristics of a transverse orthogonal anisotropic material. Each of its engineering elastic parameters satisfies Eq. (4).

$${\mathrm{E}}_{\rm{X}}={\mathrm{E}}_{\rm{Y}}$$

$${G}_{YZ}={G}_{XZ}$$

$${\mu }_{YZ}={\mu }_{XZ}$$

4 $${G}_{XY}=\frac{{E}_{X}}{2(1+{\mu }_{XY})}$$

#### Model building environment

1. Build process
  The construction of the stalk model was generated by the 3D modelling software Creo. 3D models were imported into HyperMesh for the meshing and material definition of the stalk. HyperMesh software is a powerful CAE software package with powerful pre-processing functions for finite element meshing, it can improve the quality and efficiency of the finite element analysis work. LS-DYNA is a general purpose display dynamics analysis program, capable of performing non-linear dynamics analysis, and can be used for scenarios such as collision and impact analysis, contact analysis, deformation and failure analysis, etc. Analytical solutions for finite element simulations are performed by LS-DYNA. Post-processing and analysis of results is carried out by LS-PrePost software.
2. Model building
  The simulation model was pre-processed using HyperMesh software: fix the two parts of the stalk by setting up an ‘equivalence’ operation between the phloem and the xylem. The radial direction of the ramie stalk is isotropic, the material was defined as anisotropic (mat_orthotropic elastic), the material parameters of the X–Y section are the same; Set the stalk material to MATL2, the indenter and base to MATL20 and define the material properties as (1) solid; the mesh type was set to hexahedral with a grid size of 0.5 mm. The indenter and the base are rigid bodies, its grid size does not affect the results. To reduce the amount of calculations, the grid size was set to 2.5 mm.The material parameters were set as shown in Table 3.

**Table 3:** Model parameter settings.

Parameter type	Value	Parameter type	Value
Xylem inner diameter	7.28 mm	Stalk diameter	14.23 mm
Xylem outer diameter	12.84 mm	Phloem thickness	0.67 mm
Xylem density	0.95 g/cm^3^	Phloem density	0.97 g/cm^3^
Modulus of elasticity of xylem: E_X2_, E_Y2_	7.67 MPa	Modulus of elasticity of bast: E_X1_ , E_Y1_	2.27 MPa
Modulus of elasticity of xylem: E_Z2_	320 MPa	Modulus of elasticity of bast: E_Z1_	1919.08 MPa
Xylem shear modulus G_XY_	2.95 MPa	Saprophytic shear modulus G_XY_	3.09 MPa
Xylem shear modulus: G_XZ_, G_YZ_	69.0 MPa	Saprophytic shear modulus: G_XZ_, G_YZ_	33.80 MPa
Xylem Poisson's ratio: μ_XZ_, μ_YZ_	0.04	Saprophytic Poisson's ratio: μ_XZ_, μ_YZ_	0.004
Phloem Poisson's ratio: μ_XY_	0.2 ~ 0.4	Xylem Poisson's ratio: μ_XY_	0.2–0.4
Stalk-steel static friction coefficient	0.59	Stalk-steel rolling friction factor	0.05–0.15
3. Definition of contact
  The relationship between the internal bast and xylem of the stalk has been set to fixed in the previous operation, so that the contact during bending exists mainly between the contact of the bast with the indenter and the contact of the bast with the base ① and ②. When creating the contact, the ‘contact form’ was defined as ‘SurfaceToSurfce’, ‘The Options’ was set to ‘Automatic’, select the two planes where contact will occur and set a reasonable coefficient of dynamic and static friction, the former determined by the experimental design plan and the latter measured by friction test.
4. Constraint addition
  The following constraints have been added to the model. The two supports are fixed rigid bodies, the indenter model is constrained in the displacement X and Z directions, rotation is constrained in the X, Y and Z directions. The indenter was loaded in the -Y direction, the indenter displacement was set to 14 mm. The indenter moves downward to bend and deform the straw after contacting it. The deformation curve of the force was measured by a universal material testing machine. The stalk was in an unconstrained state, so there was relative sliding between it and the three rigid bodies.The overall model is shown in the Fig. 8.

**Figure 8:**
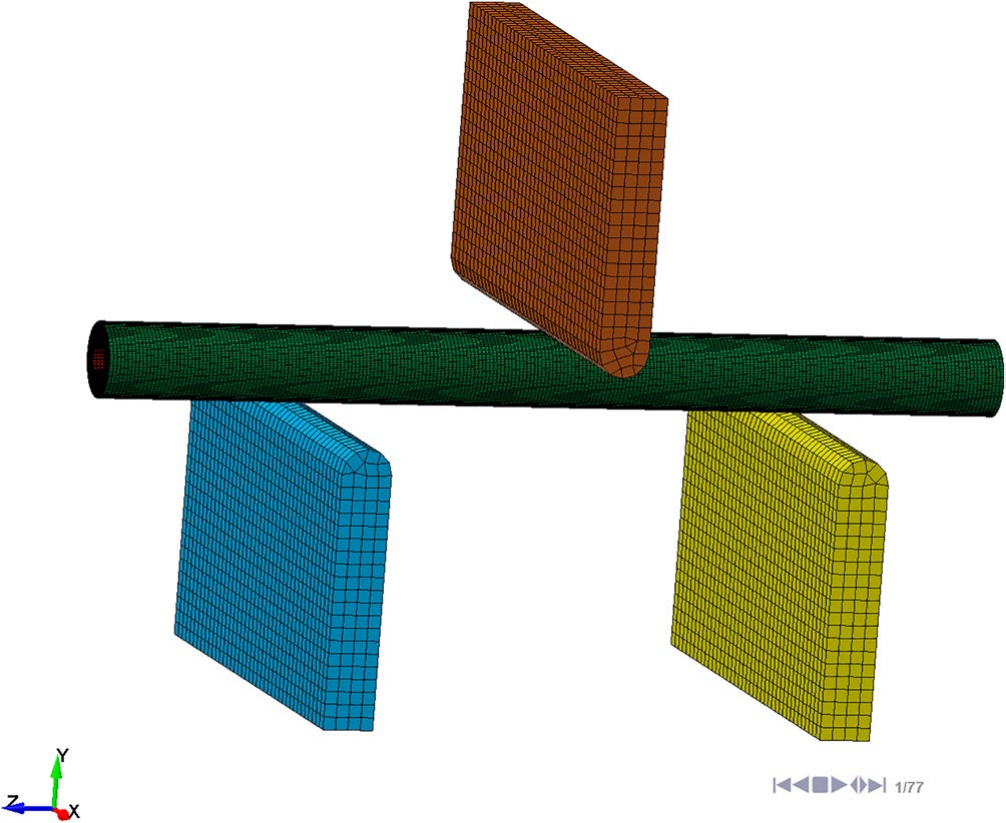
Three-point bending mesh model.

#### Parameter calibration process

Combined with the force state of straw in the actual conveying process, it is believed that its bending deformation is mainly caused by the three-point force. Therefore, a three-point bending test is used in this paper.

1. Method of parameter calibration
  ① Design-Expert 13 software was applied to design the experiments, using Creo, HyperMesh, LS-DYNA and LS-PrePost for 3D model building, material definition, meshing, solving and post-processing. ② Parameter optimisation solving by central combination tests to determine the material and contact parameters of the stalk model. ③ The model was established with reference to the single-factor test data, and the difference between the actual and simulated values of the stalk bending force was compared to verify the accuracy of the material and contact parameters.
2. Central composite experiment design
  Rolling friction coefficient, xylem and phloem Poisson's ratio are three parameters of ramie stalk material, and their value range can be determined by consulting the data. Therefore, a ramie stalk model was established for a central combination design test, with the bending damage force as the index, to obtain a regression model between the maximum damage force Y and the rolling friction coefficient X_1_ , the bast Poisson's ratio X_2_ and the woody Poisson's ratio X_3_. Solve the regression equation to get the optimal parameter combination of the model. The factor level coding table is shown in Table 4.

**Table 4:** Central composite design factor coding.

Factor Level	Parameter		
	Rolling friction coefficient X_1_	Phloem Poisson's ratio X_2_	Xylem Poisson's ratio X_3_
1.682	0.15	0.4	0.4
1	0.13	0.36	0.36
0	0.1	0.3	0.3
−1	0.07	0.24	0.24
−1.682	0.05	0.2	0.2

### Ethics statement

I have permission to collect the ramie. Collection of plant material, must comply with relevant institutional, national, and international guidelines and legislation.

## Result

### The result of ramie bending test

**Figure 9:**
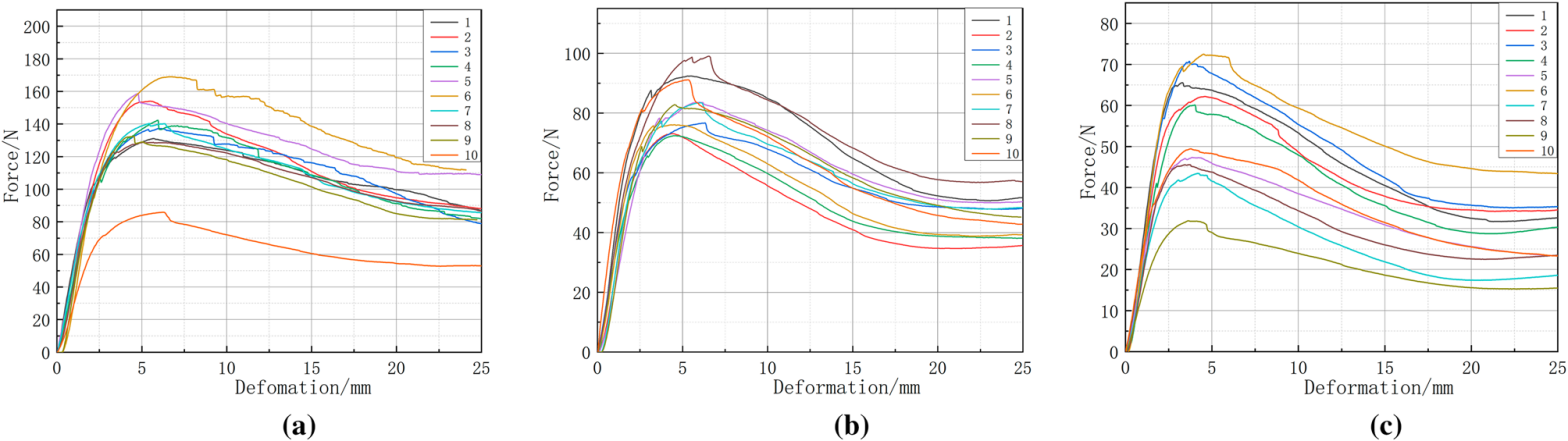
Force–deformation curve of 'loading position' single factortest. (**a**) 10–28 cm, (**b**) 60–78 cm, (**c**) 110–128 cm.

Sampling tests were carried out at three positions from the ground: 10–28 cm, 60–78 cm, and 110–128 cm, ten groups were taken at each level for a total of 30 tests. The Force–Deformation curve is shown in Fig. 9.

The mean Bending modulus of elasticity of the whole stalk at the three loading locations was 47.88 MPa, 40.06 MPa and 32.70 MPa, with standard deviations of 5.01 MPa, 5.51 MPa and 4.18 MPa. The mean values of the maximum forces when failure occurred were 132.37 N, 84.8 N, and 54.87 N, and the standard deviations were 5.01 N, 5.51 N, and 4.08 N, respectively.

### The results of parameter calibration

#### Results of central composite design experiment

On the basis of the bending test, a central composite design was carried out for the finite element bending simulation test of the stalk. The experimental design and results are shown in Table 5.

**Table 5:** Central composite design scheme and results.

NO	X1	X2	X3	Maximum destructive force Y/N
1	0	0	0	84.3
2	0	0	1.682	85.8
3	0	0	0	84.7
4	1.682	0	0	84.7
5	1	1	1	85.5
6	−1	−1	−1	83
7	0	0	0	84
8	1	−1	−1	83.4
9	0	0	0	84.6
10	0	0	0	84.4
11	0	0	0	84.5
12	-1	1	1	85.5
13	0	0	0	85.3
14	-1	−1	−1	84.9
15	1	−1	−1	84
16	1	1	1	85
17	0	−1.682	−1.682	82.9
18	−1	1	1	84.8
19	−1.682	0	0	84.5
20	0	0	0	84.4

#### Regression equation building and significance analysis

The ANOVA of the test results in Table 6 showed a good fit of the whole model (P < 0.001). X_2_, X_3_, X_1_ X_2_ and X_2_ X_3_ had a very significant effect on the maximum bending force, which indicated that both were key factors in the response surface design. X_1_ had P > 0.05, indicating that this factor did not affect the index. The coefficient of determination of the model is 0.95, indicating that the regression model is consistent with the actual results. The amount of misfit was 0.18 > 0.05, indicating that the test produced very little error, and excluding the non-significant term, the results are shown in Table 6.

**Table 6:** ANOVA of central composite design experiment.

Source	Sum of squares	df	Mean square	F-value	P-value
Model	10.98	6	1.83	45.57	< 0.0001
X2	2.54	1	2.54	63.2	< 0.0001
X3	7.89	1	7.89	169.41	< 0.0001
X1X2	0.28	1	0.28	7.01	0.02
X2X3	0.21	1	0.21	9.69	0.0082
Residual	0.52	13	0.052		
Lack of fit	0.41	8	0.022	2.39	0.18
Pure error	0.11	5	0.022		
Core total	11.5	19			

Taking the maximum bending failure force as the target value, the Design-Expert software was used for analysis, and the regression equation of the maximum bending force of the ramie stalk was obtained:

5 $$\mathrm{Y}=84.51+0.43{\mathrm{X}}_{2}+{0.76\mathrm{X}}_{3}-0.19{\mathrm{X}}_{1}{\mathrm{X}}_{2}-\mathrm{O}.16{\mathrm{X}}_{2}{\mathrm{X}}_{3}$$

Based on the test results of the single factor test, the optimal combination of parameters X_1_, X_2_, and X_3_ was obtained by solving the regression equation. The results of the model parameters are shown in Table 7.

**Table 7:** Test results.

Parameter	Value
Coefficient of dynamic friction	0.12
Phloem Poisson's ratio	0.29
Xylem Poisson's ratio	0.32

#### Parameter validation

In order to ensure the feasibility, accuracy and general applicability of the model parameters, We selected three sets of representative test curve data from the single-factor ‘loading positions’ experiment, built models for simulation analysis. The results of the validation tests are shown in Table 8. The maximum bending failure force error between the simulated values and the actual test results is less than 5%. In addition, the maximum bending damage force showed a good linear relationship with the stalk diameter, indicating that the parameter calibration method is correct and the finite element model developed is accurate and useful.

**Table 8:** Validation results.

Outer diameter (mm)	Inner diameter (mm)	Phloem thickness (mm)	Actual value (N)	Simulation value (N)	Error (%)
15.1	7.5	0.72	92.42	96.20	4.11
14.3	7.45	0.64	76.69	78.65	2.56
14.7	7.5	0.65	83.44	87.40	4.75

### Stalk bending principle

**Figure 10:**
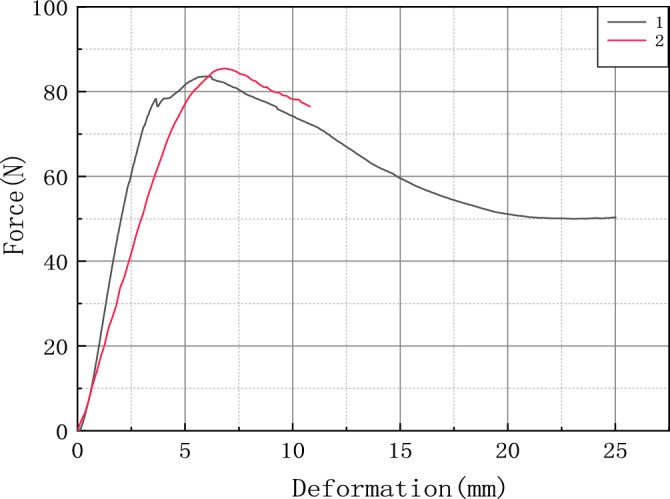
Comparison of simulation test and real test.

The force–deformation curve of the bending test is shown in Fig. 10, the red curve 1 represents the real test, and the black curve 2 is the finite element simulation test. By comparing the finite element simulation with the actual test, it can be seen that the force–deformation curve is almost the same, showing a linear relationship in the elastic stage, following Hooke's law, the deformation can be recovered when the force is removed. When the bending force reaches the maximum value, then decrease, and the stalk is damaged.

**Figure 11:**
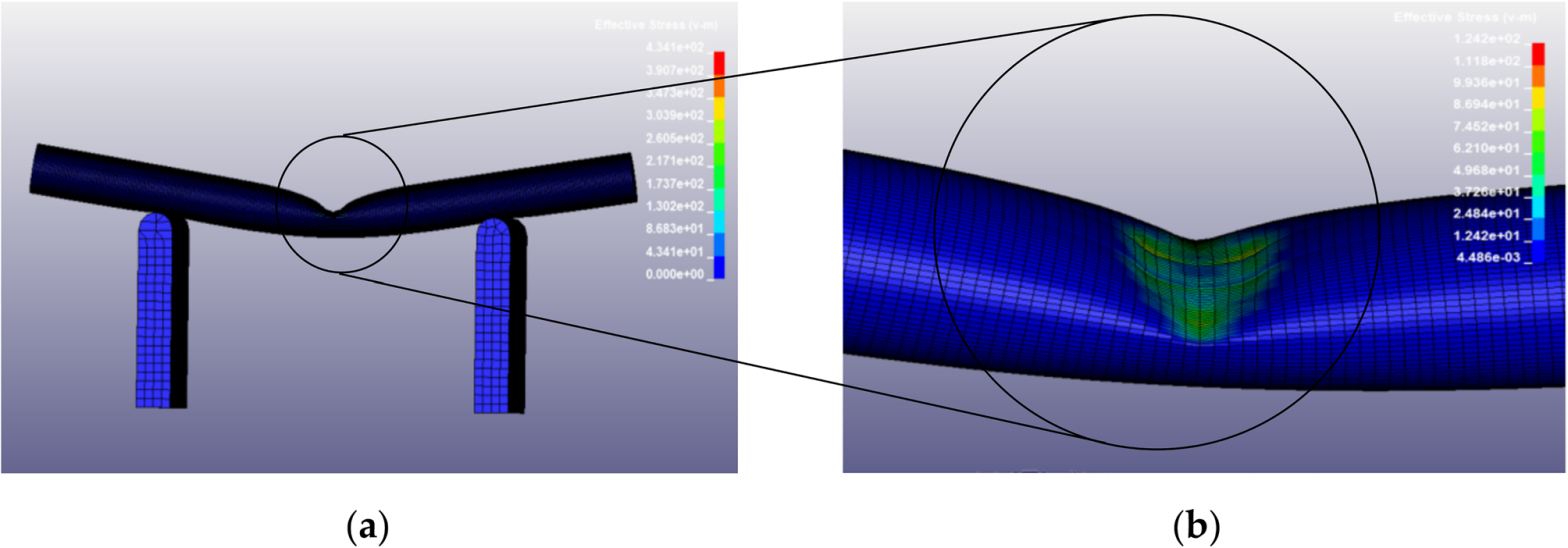
Stress distribution during bending. (**a**) Overall deformation and force diagram. (**b**) Force diagram of the contact surface between the indenter and the stalk.

The deformation and stress distribution of the ramie stalk in bending is shown in the Fig. 11. When subjected to vertical downward load, the upper section of the stalk is under pressure, and the stress is mainly concentrated on the contact surface between the indenter and the stalk. The lower section of the stalk is under tension, the rounded bottom surface of the outer part of the bend is deformed into an oval shape due to the tensile properties of the ramie fibre^21^.

**Figure 12:**
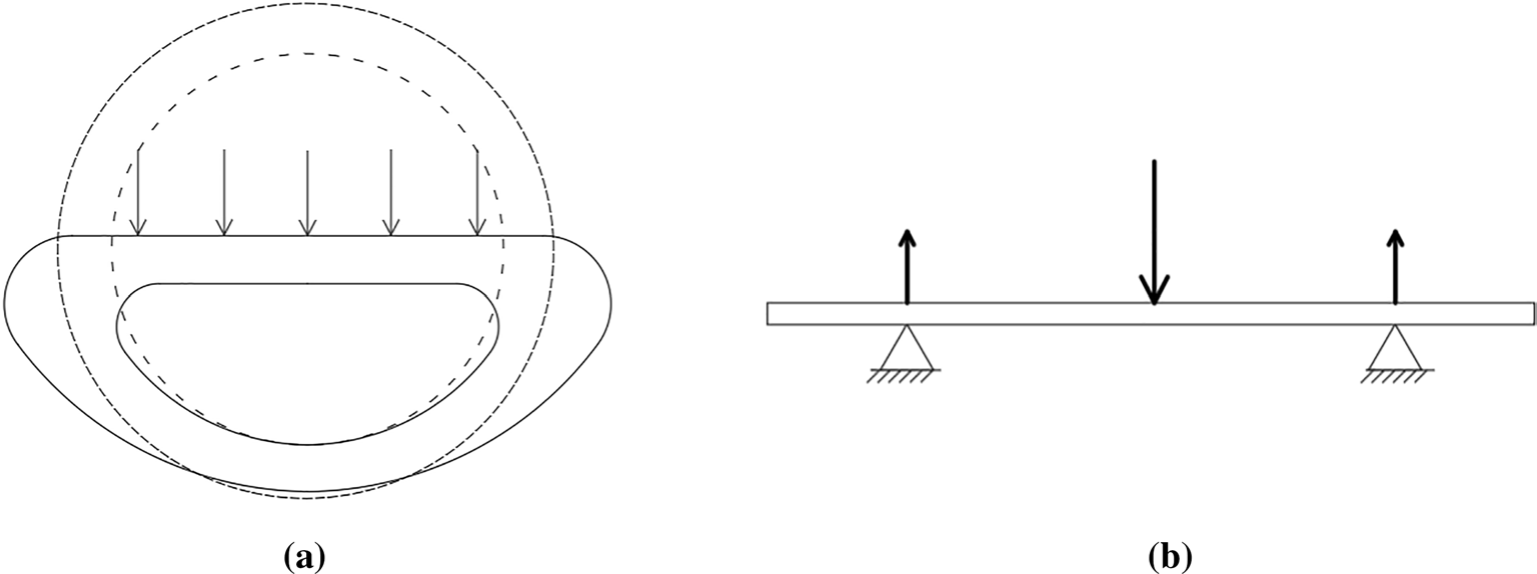
Force and deformation figure. (**a**) Stress and deformation of the cross section of the stalk; (**b**) lateral force diagram.

According to the mechanics of materials theory^22^,from the formula for calculating the maximum positive stress strength, the formula for the moment of inertia and the formula for the flexural section coefficient (5–6), it can be concluded from the analysis that the moment of inertia and the flexural section coefficient of the stalk change as a result of the large deformation of the stalk cross-section under stress, which also can be interpreted as a breakdown of the original stable structure of the stalk. Compared to a purely bending force regime,The existence of lateral force resulted in a significant decrease in the overall bending strength of the stalk.The deformation of the cross section of the stalk and the overall forces are shown in Fig. 12.

6 $${\sigma }_{max}=\frac{{M}_{max}{y}_{max}}{{I}_{z}}=\frac{{M}_{max}}{W}$$

7 $$\mathrm{W}=\frac{{\mathrm{I}}_{\rm{z}}}{\mathrm{y}}=\frac{\uppi \left({\mathrm{D}}^{4}-{\mathrm{d}}^{4}\right)}{32\mathrm{D}}$$

where σ_max_ is maximum positive stress, M_max_ is bending moment, D is outer diameter, d is inner diameter, I_Z_ is moment of inertia of the cross section against the neutral axis, W is bending section coefficient.

## Discussion

1. Bending analysis
  Obataya et al.^23^ explored the bending mechanical properties of bamboo, and found that the flexibility of bamboo is due to the combined effect of the large compressive deformation allowed by the inner xylem and the ability of the outer bamboo fibers to withstand compressive stress, and analyzed the effect of the internal fiber-foam composite structure on the overall Influence of flexibility and flexural ductility. Compared with bamboo, the moisture content of the xylem of ramie reaches 80%, and the xylem is fluffy and easily deformed, so when the stalk is subjected to lateral force, the xylem cannot play a supporting role.
  Combined with the stress distribution and deformation failure curve obtained from the simulation results, the failure deformation of this stress method is different from the pure bending stress of the stalk. When bending occurs, the lower half surface of the stalk is stretched in the Z-direction, and the lower half surface tends to be flat due to the protective effect of the bast fiber; when the force is applied in the Y-direction, the bending resistance coefficient of the stalk decreases continuously because the xylem is fluffy and easily deformed, and the upper section is constantly depressed, and the bending resistance performance of the stalk is greatly reduced.
2. Finite element analysis environment
  This paper is completed by a combination of multiple software. HyperMesh software is called "pre-processing expert", and is used to complete pre-processing work such as mesh division and material definition, this software has strong mesh processing capabilities. the model establishes a hexahedral mesh, which can better fit the surface, and the overall simulation test has a good degree of reduction.Properly increasing the volume of the mechanical structure (rigid body) unit body can reduce the amount of simulation calculation. Although the size and number of meshes will affect the simulation accuracy to a certain extent, the overall impact on the overall results of the test is small. LS-DYNA software specialises in the analytical solution of collision and impact problems, and in the actual harvesting environment, the stalk and the mechanical structure usually come into contact in the form of collision, so the model built in this way can lay the foundation for the subsequent analysis of rigid body-stalk collision studies.
  As an effective and mature numerical calculation method, DEM has been gradually applied in the study of agricultural mechanisation to analyse the interaction between machinery and discrete unitary bodies, It is a digital research method for discrete body motion^24^. The addition of parameters such as mechanics and material properties enables the creation of a parametric discrete element model, so that the model of the stalk can be parametrically calibrated using EDEM analysis software as well. When Shi et al.^25^ studied the work of straw crushing, they established a finite element model of straw straw by discrete element method (DEM) and EDEM software, which was used for the analysis of straw crushing and laying process . Song et al.^26^ developed a simulation model of a seeder anti-clogging device, including soil, straw and mechanical structures, and calibrated the physical properties of the slab caked black soil clods and wheat straw.
  Edem software can quickly and efficiently generate a collection of particles according to the shape of the object. In this software, the first step is usually to define the spheres that need to fill the model, and use a variety of sphere particle combinations to fill irregular objects. The spherical granular units are too regular, the surface formed by the accumulation of small spheres cannot fully fit the contours of the object, the friction coefficient is far from that of the real surface, and the collision and rolling characteristics are different compared to the real one. Adjusting the size and number of filled particles can improve the error, but will greatly increase the amount of calculation^27^. For example, in the small wheat grain model established by Song Chen et al.^28^ the difference in the way the model is filled causes an error of 10.97%, so the method is suitable for discrete granular materials.
3. Parameter calibration scheme
  The calibration of the material parameters means that before the simulation can be carried out, a large number of experiments must be carried out on the material to be used in the working conditions to obtain the relevant data. The basic test procedure for the calibration of the model parameters is to first obtain the significant factors affecting the mechanical properties through a two-level factorial test, and then to establish the regression equation and solve for the significant factors through a regression test or a central combination test to obtain the optimal combination of parameters^12,26^.
  By measuring and consulting data, the ramie stalk bending model has only three parameters that cannot be measured accurately. Therefore, the paper simplifies the screening process and only requires a central combination design to obtain the optimal parameter solution to obtain all the parameters of the model, and most of the parameters are measured by experiments, which results in less experimental error. In the parameter validation test, the modeling simulation test is based on the previous single-factor bending test, and multiple sets of single-factor data are taken to verify the accuracy of the model.The result parameter verification method is more reasonable and convincing.

## Conclusions

1. Many blank parameters of ramie field such as moisture content, density, diameter, xylem and bast wall thickness, and static friction coefficient were obtained. The bending modulus of elasticity and fiber to stalk ratio of ramie stalks were measured and calculated. The single-factor bending tests showed that the loading position had a significant effect on the bending mechanical properties of the stalks, and the bending strength gradually decreased as the stalk height increased.
2. Finite element bending simulations of ramie stalks were conducted to analyze the bending deformation process and force distribution. The poor bending resistance of ramie stalks was analyzed: the ramie woody part has a hard texture for support, and the fiber tensile strength is high, which has an enhanced effect on the overall bending strength. However, due to the hollow structure, the stalks are first compressively deformed during bending, which reduces the cross-sectional bending resistance coefficient and leads to a lower bending resistance.
3. A central combination design simulation test was proposed to calibrate the key parameters of the ramie model, and finally the best combination of dynamic friction coefficient, wood Poisson's ratio and toughness section was obtained. The model parameters were as follows: rolling friction coefficient 0.12, Poisson's ratio of bast 0.29, and Poisson's ratio of wood 0.32. Validation tests were conducted on the simulation results, and the final error was within 5%. It proved that the calibration parameters were valid and the established bending model was accurate and reliable.

Through the mechanical tests and calibration tests, the complete basic parameters of ramie stems were obtained, filling the gap of related research, and the established bending mechanics model was used as a reference for subsequent simulation studies on ramie harvesting technology. The calibration and validation methods adopted in this paper can be applied to simulation studies of other long-stemmed crops.

## Data Availability

The datasets used and/or analysed during the current study available from the corresponding author on reasonable request.
